# Surveillance of important bacterial and parasitic infections in Danish wild boars (*Sus scrofa*)

**DOI:** 10.1186/s13028-020-00539-x

**Published:** 2020-08-03

**Authors:** Heidi Huus Petersen, Nao Takeuchi-Storm, Heidi Larsen Enemark, Stine Thorsø Nielsen, Gitte Larsen, Mariann Chriél

**Affiliations:** 1 Centre for Diagnostic, Technical University of Denmark, Kemitorvet, 2800 Kgs. Lyngby, Denmark; 2 Department of Animal Health and Food Safety, Norwegian Veterinary Institute, Sentrum, P.O. Box 750, 0106 Oslo, Norway; 3 Centre for Rich Nature, The Danish Environmental Protection Agency, Tolderlundsvej 5, DK-5000 Odense C, Denmark

**Keywords:** *Brucella*, Gastrointestinal parasites, *Metastrongylus*, MRSA, *Salmonella*, *Trichinella*, Wild boars

## Abstract

**Background:**

Similar to the situation in other European countries, Danish wild boars may harbour a wide range of pathogens infectious to humans and domestic pigs. Although wild boars must be kept behind fences in Denmark, hunting and consumption of the meat may cause zoonotic transmission. Moreover, most infections of wild boars are transmissible to domestic pigs, which may have important economic consequences. The aim of this study was to investigate whether Danish wild boars were infected with bacteria and parasites transmissible to humans or domestic pigs: *Brucella suis*, methicillin-resistant *Staphylococcus aureus* (MRSA), *Salmonella* spp., *Trichinella* spp., lungworms and gastrointestinal parasites, especially *Ascaris suum*. This is the first study to investigate the prevalence of these important pathogens in Danish wild boars.

**Results:**

Wild boars from eight enclosures were analysed over a 5-year period. All tested wild boars were negative for *B. suis* (n = 240), MRSA (n = 244), *Salmonella* spp. (n = 115) and *Trichinella* spp. (n = 232), while eight parasite genera were identified in the faeces (n = 254): *Ascaris suum, Capillaria* sp., *Cystoisospora suis, Eimeria* spp., *Metastrongylus* sp. (lungworm), *Strongyloides ransomi*, *Trichuris suis* and strongylid eggs, i.e. strongyles not identified to the genera. *Eimeria* spp. and *Metastrongylus* sp. had the highest prevalence (92.3 and 79.5%, respectively) and were identified in wild boars from all eight enclosures, while the remaining parasite genera were present more sporadically.

**Conclusions:**

Wild boars from Denmark constitute a low risk of transmitting *B. suis*, MRSA, *Salmonella* spp. and *Trichinella* spp. to humans or domestic pigs, while economically important parasites transmissible to domestic pigs are highly prevalent in the wild boar population.

## Background

Wild boars (*Sus scrofa*) have an extensive worldwide distribution, and a significant increase in their population was observed in Europe in the last decades [1]. Wild boars have one of the largest geographical ranges of all terrestrial wild animal species [2]. In Denmark, free-living wild boars were extinguished in 1801 [3], and today, wild boars must be kept behind fences. In the Danish Central Livestock Register, 53 enclosures are currently listed for keeping wild boars [4], and officially, the wild boar population consist of 654 animals. However, the actual number is unknown. The enclosures include natural habitats up to 40 km^2^, where the wild boars are kept for hunting or farming. Free-living wild boars are sporadically reported, when they escape from enclosures or cross the border from Germany. However, hunters are encouraged to shoot these free-living wild boars all year round. In December 2019, a fence (1.5 m high and 70 km long) was built at the German–Danish border to prevent wild boars from crossing the Danish land border from Germany.

In general, wild boars are considered a potential risk for transmission of severe pathogens to humans and domestic pigs. Humans can come in contact with live or dead wild boars and become infected e.g. during hunting, recreational use of wild boar habitats or through consumption of wild boar meat, or exposure to the escaped wild boars. Moreover, humans or wild boars can transfer infections to domestic pigs [5–8]. Currently, domestic pigs from Denmark are free from important notifiable diseases such as classical- and African swine fever, Aujeszky’s disease, brucellosis and trichinellosis [9], but the risk of disease transmission from wild boars to domestic pigs is likely to increase due to the trend of converting pig production from indoor to outdoor housing. Pig production and export of pork meat is a major and important industry in Denmark with a production of around 20 million slaughter pigs per year [10]. Although the Danish wild boars are confined in enclosures, other wild animals can function as reservoir hosts for transmission of pathogens from wild boars to domestic swine, e.g. brown hares (*Lepus europaeus*) infected with *Brucella suis* [11] and rodents infected with *Trichinella* spp. [12].

This study focused on the bacteria and parasites, which in Denmark currently are considered most important to the pig industry and public health, i.e. *B. suis*, *Salmonella* spp., methicillin-resistant *Staphylococcus aureus* (MRSA), *Trichinella* spp., and various gastrointestinal parasites and lungworms.

The genus *Brucella* is a group of zoonotic bacteria causing brucellosis. *Brucella* spp. are infectious to several mammals including cattle, sheep, goats, pigs, and humans. In pigs, brucellosis caused by infection with *B. suis* manifests as abortion, orchitis, and lameness [13], while in humans, the disease has varying clinical manifestations, where the acute stage is characterized by nonspecific symptoms similar to a flu-like or septicaemic illness [14]. Brucellosis has not been detected in Danish pig herds since 1994 and 1999, where *B. suis* biovar 2 have been found in free-ranging pig herds [15].

*Salmonella* are widespread bacterial pathogens shared by humans, domestic- and wild animals including wild boars [16–18]. Human foodborne *Salmonella* outbreaks are common in Europe [19]. Wild boars can harbour several different serovars of *Salmonella* including *S. typhimurium* and *S. enteritidis* [17], both of which cause numerous salmonellosis cases in humans annually [19]. In humans, salmonellosis can cause severe disease and even death, while animals are often asymptomatic carriers of the infection. *Salmonella* infections occur in Danish pigs [20] as well as in wild animals including e.g. hedgehogs (*Erinaceus europaeus*), badgers (*Meles meles*) and harbour seals (*Phoca vitulina*) [21].

Lately, MRSA has become a problematic microorganism because of its resistance to almost all beta-lactams and the ability to transfer the resistance mechanism to other non-beta-lactamase producing pathogens. Previously, MRSA was a pathogen mostly associated with hospitals, but also people without contact to hospital environments are widely diagnosed with MRSA. MRSA has been recorded from Danish pigs [22], from retail meat [23], farmed mink [24], and horses [25]. Studies on MRSA in wild boars are currently scarce, but MRSA has been identified in wild boars from Germany and Spain [26, 27].

Denmark is officially recognised as a region with negligible risk of *Trichinella* in farmed pigs [28]. Currently, no systematic surveillance of *Trichinella* spp. in Danish wildlife occur, but previous surveillance projects have demonstrated presence of *T. pseudospiralis* in wild mink on the Island of Bornholm in 2007 (Data not published), and three cases of *Trichinella* spp. in red foxes (0.1%) in the mid-1990s [29]. Therefore, Danish wildlife, including wild boars, could potentially be infected with *Trichinella* spp.. Currently, all wild boars shot in Denmark or shot abroad and imported to Denmark for consumption must be tested for *Trichinella* spp.. Wild boars are asymptomatic carriers of *Trichinella* spp., while the symptoms of trichinellosis in humans include mild non-bloody diarrhoea, nausea, vomiting, abdominal discomfort, persistent fever, sweating, chills, periorbital oedema, urticarial rash, and conjunctival or splinter and ungual haemorrhages [30].

Besides *Trichinella* spp., wild boars can harbour many parasites of zoonotic and economic importance. Gastrointestinal parasites and lungworms are commonly detected in European wild boars [31–34], and they can infect domestic pigs and vice versa. Parasitic infections in domestic pigs are commonly subclinical, but weight loss, diarrhoea, reduced growth, depression, fatalities, reduced carcass quality and chronic and paroxysmal coughing can appear [35–38], depending on the parasite genera and infection dose. Hence, parasitic infections may cause significant economic losses to the pig production. Additionally, the helminths *A. suum* and *Metastrongylus* are zoonotic [39–41] and the same may be true for *Trichuris suis* [35]. In humans, *A. suum* infection may cause severe visceral larval migrans causing liver and lung lesions [42].

The aim of this study was to investigate the prevalence of *B. suis*, MRSA, *Salmonella* spp., *Trichinella* spp., gastrointestinal parasites and lungworms in wild boars from Denmark to assess if they pose a risk of transmitting these pathogens to humans and domestic pigs. Surveillance of wild boars for African- and classical swine fever as well as Aujeszky’s disease is mandatory in Denmark and testing for these pathogens were therefore not included in this study.

## Methods

### Study area and sample collection

**Fig. 1:**
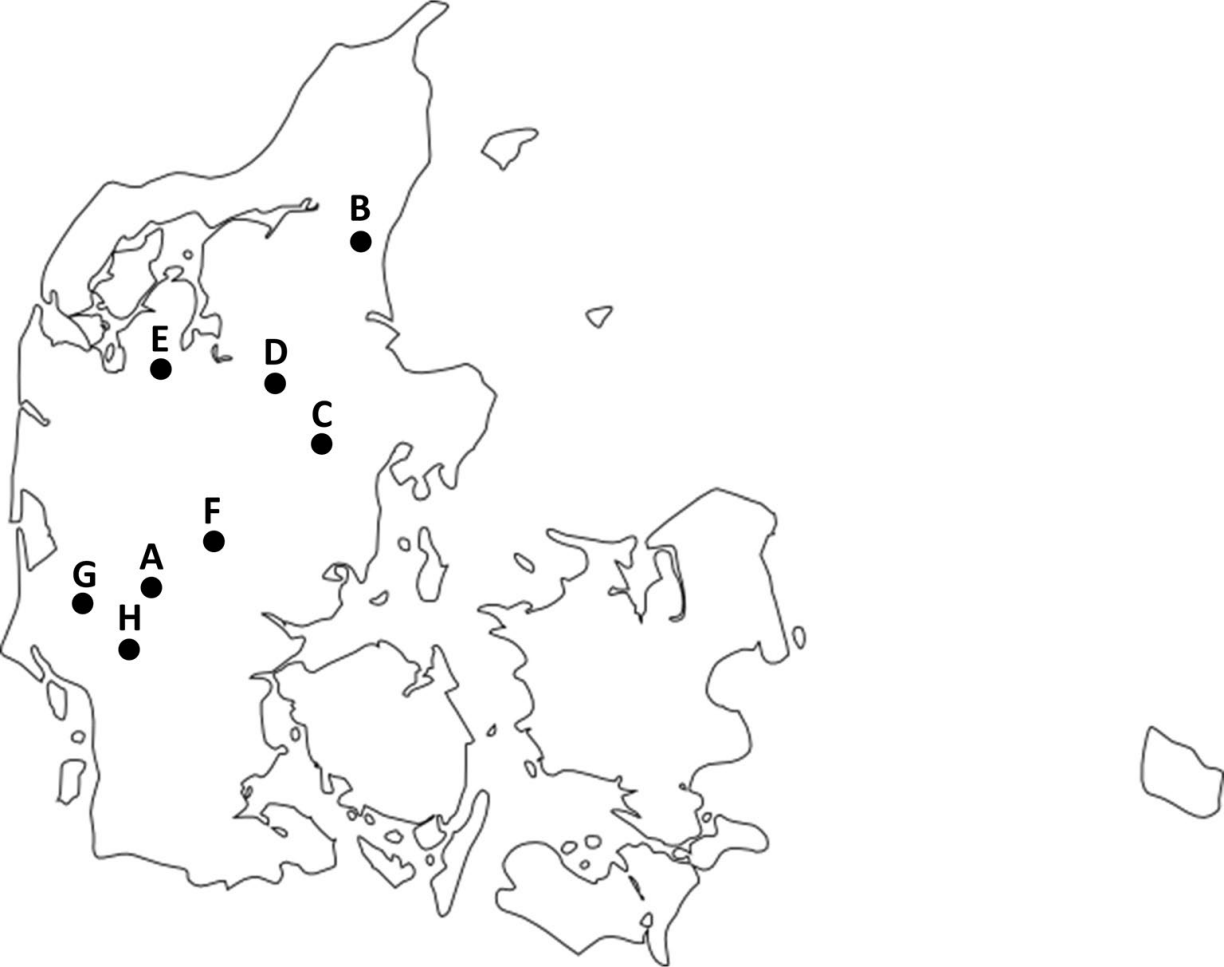
Map of Denmark showing the geographical origin of the wild boar enclosures included in the study

The Danish Veterinary and Food Administration funded the study, and decided on the yearly sample size. The Veterinary Institute (now Centre for Diagnostic), Technical University of Denmark selected the study enclosures based on the size of the actual enclosure and the willingness of the owners to participate. The aim was to sample five large enclosures per hunting season. Since the sampling was based on voluntary participation by hunters, this was not always possible (Table 1). Altogether, eight separate outdoor enclosures in mainland Denmark were included in the study (Fig. 1).

**Table 1:** Overview of wild boars included in the study according to hunting seasons and enclosure

Enclosure	Hunting season					
	2014/2015	2015/2016	2016/2017	2017/2018	2018/2019	Total
A	8		21	10	16	55
B	19		19	15	14	67
C	10		7	12	10	39
D	11			10	12	33
E	11					11
F		11		14		25
G					15	15
H		10				10
Total	59	21	47	61	67	255

The enclosures are natural habitats with forest, lakes and/or moor covering up to 40 km^2^. The wild boars were typically fed supplemental feed at permanent feeding sites or on the ground. If severe clinical disease was observed, the animals were euthanized for welfare reasons. The sampling took place during the hunting seasons (1st October to 31st January) 2014/2015 to 2018/2019. Each enclosure was sampled once during each hunting season. The samples were collected during one to four hunting seasons from each enclosure (Table 1). No animals were hunted for the purpose of this study.

Between seven and 20 wild boars were sampled per sampling day and enclosure (Table 1). Faecal samples from rectum (min. 30 g), diaphragm (approx. 5 × 5 cm), uterus or one testicle, spleen (approx. 5 × 5 cm) and nose swabs were collected immediately post mortem and transported in cooling boxes directly to the laboratory for analyses.

### Analysis

Table 2 shows the number of wild boars analysed for each pathogen. Some samples were unsuited for analyses due to severe tissue damage after passage of the bullet. If possible, the approximate age was assessed by the hunters and recorded for each animal.

**Table 2:** Overview of the number of wild boars in Denmark (2014–2019) examined for pathogens that can be transferred between wild boars, domestic pigs and humans

Pathogen	Number of wild boars analysed
*Brucella* spp.	240
Parasite eggs/oocysts	254
Methicillin-resistant *Staphylococcus aureus* (MRSA)	244
*Salmonella* spp.	115^a^
*Trichinella* spp.	232

^a^Wild boars sampled in in the hunting seasons 2017/2018 and 2018/2019 were not analysed for *Salmonella* spp.

The diaphragms were analysed for *Trichinella* spp. larvae by the magnetic stirrer method for pooled sample digestion according to Mayer-Scholl et al. [43]. Briefly, individual diaphragm samples (20 g from each animal from up to 10 animals from the same enclosure) were mixed and digestion fluid was added. The mixture was then stirred for a maximum of 60 min at 45 °C, subsequently sieved and allowed to sediment for 30 min. The sediment was collected and allowed to sediment for another 10 min. The supernatant was removed and the sediment was analysed for presence of *Trichinella* spp*.* larvae by stereomicroscopy at ×20 magnification.

The faecal samples were analysed for parasite eggs/oocysts and *Salmonella* spp.. Parasite eggs/oocysts were quantified using the modified McMaster technique [44] with a sensitivity of 5 eggs/oocysts per g faeces. Briefly, 4 g of faeces was suspended in 56 mL tap water, sieved through gauze, and 10 mL was centrifuged at 178 × *g* for 10 min. The supernatant was removed, the pellet was re-suspended in 3 mL of flotation fluid (saturated saline with glucose, 50 g/100 mL, specific gravity 1.27 g/mL) and a disposable McMaster chamber was filled with the mixture. The McMaster disposable chambers were prepared in our laboratory by gluing two small glass slides (38 × 26 × 1 mm) onto a larger glass slide (76 × 38 × 1 mm) separated by 24 mm space, and finally, a microscopic cover glass (50 × 25 × 0.2 mm) was glued onto the two small glass slides to create a counting chamber holding 0.6 mL. All eggs/oocysts in the whole chamber were identified by microscopy at ×100 magnification.

*Salmonella* spp. were analysed by faecal culture; 225 mL peptone buffer (Oxoid, Hampshire, UK) was added to 25 g of faeces in a plastic cup, homogenised by stirring and incubated at 37 °C for 16–20 h. Following incubation, 1 mL of the faecal mixture was collected; three drops of the mixture was inoculated onto Modified Semisolid Rappaport Vassiliadis agar (MSRV) (Oxoid, Hampshire, UK), while the remaining was added to 9 mL of Selenit broth (Oxoid, Hampshire, UK). Both were incubated for 18–24 h at 41.5 °C. Suspected *Salmonella* spp. colonies from the MSRV agar plate and 10 µL of the Selenit broth were plated onto a Xylose Lysine Deoxycholate agar (Oxoid, Hampshire, UK) and a Brilliant Green Agar (Oxoid, Hampshire, UK) and incubated at 37 °C in 18–24 h.

The spleen and the genital organs were analysed for *B. suis* by culture. The spleen and genital tissues (2 g of each) were pooled, 2.5 mL sterile water was added and the sample was homogenised for 2 min in a Stomacher® laboratory blender. Two drops of the resulting broth were then inoculated onto *Brucella* agar plates and incubated at 37 °C with CO_2_ for a maximum of 10 days.

Presence of MRSA was investigated by adding 3–4 mL Mueller–Hinton bouillon with 6.5% NaCl (SSI Diagnostica, Hillerød, Denmark) to a 15 mL tube containing the nasal swab, followed by incubation on a shaker table at 37 °C for 24 h. A loopful of broth was inoculated onto a MRSA 2 agar (Oxoid, Hampshire, UK) and incubated at 37 °C for 24–48 h, following which suspected colonies were subcultured onto a C-cattle blood agar (SSI Diagnostica, Hillerød, Denmark).

### Data analysis

The prevalence and the 95% confidence intervals (CI) were calculated for each parasite species for the total study population. The prevalence, median and mean intensity (95% CI), min and max FEC/FOC were also calculated for wild boars ≤1 and >1 year for each parasite species. Differences in prevalence between age groups were determined using a binary logistic regression model with the parasite genera (*Cystoisospora suis* and *Strongyloides ransomi* were not tested) as the dependent variable and age groups (≤1 or >1 year) as the independent variable. The age was not assessed for 36.1% of the wild boars, and therefore excluded from the analysis. The differences in mean *A. suum*, *Metastrongylus* sp. and strongyle faecal egg count (FEC) and *Eimeria* spp. faecal oocyst count (FOC) for wild boars positive and negative for *T. suis* were determined by a one-way ANOVA with the log-transformed FEC/FOC as dependent variable and *T. suis* infection status (positive/negative) as independent categorical variable to determine if *T. suis* positive wild boars had higher FEC and FOC than *T. suis* negative wild boars. A P-value of ≤0.05 was considered significant. All statistical analyses were done in SAS for windows version 9.4 (SAS institute Inc., Cary, NC, USA).

## Results

Altogether, 255 wild boars were sampled. Of these, 56.5% were males and 40.4% were females, while sex was not recorded for 3.1% of the wild boars. Of the wild boars where age was noted, 32.2% were ≤1 year and 31.8% were >1 year. The age was not assessed for 36.1% of the wild boars.

All tested wild boars were negative for *Trichinella* spp. larvae, *Salmonella* spp., *B. suis*, and MRSA, while parasite eggs/oocysts were identified from 0.4–92.3% of the wild boars (Table 3). The following parasite eggs/oocysts were identified: *Ascaris suum, Capillaria* sp., *C. suis, Eimeria* spp. (the oocysts were not identified to species level, but based on morphology, a mixture of different species was present), *Metastrongylus* sp., strongylids not identified to species level, *S. ransomi* and *T. suis*.

*Eimeria* spp. were the most commonly identified parasite, followed by *Metastrongylus* sp., strongyles, *Capillaria* sp., *A. suum* and *T. suis*, while *C. suis* and *S. ransomi* were identified from only one wild boar each (Table 3). *Eimeria* spp. oocysts and *Metastrongylus* sp. eggs were identified in faeces from wild boars from all eight enclosures (Table 3).

**Table 3:** Overview of parasites detected coproscopically in wild boars in Denmark in 2014–2019 (n = 254)

Parasite genera	No. of positive	Prevalence 95% CI	No. of enclosures with positive wild boars	Age ≤1^a^			Age >1^a^		
				Prevalence 95% CI	Median egg/oocyst excretion [min – max]	95% CI of mean intensity	Prevalence 95% CI	Median egg/oocyst excretion [min – max]	95% CI of mean intensity
*Eimeria* spp.	234	92.3 88.8; 95.5	8	92.1 85.2; 99.0	2760 [10−200,000]	5,021; 23,257	87.9 81.3; 84.4	7,980 [10 - 378,000]	15,884; 39,009
*Metastrongylus *sp.	202	79.5 74.5; 84.5	8	90.5 83.0; 98.0	130 [10−11,100]	429; 1,512	66.7 57.2; 76.1	83 [10 - 16,500]	184; 1,409
Strongyles	150	59.1 53.0; 65.1	7	52.4 39.7; 65.1	120 [10 – 3,900]	217; 984	56.6 46.7; 66.5	48 [10 – 1,600]	72; 250
*Capillaria *sp.	28	11.1 7.1; 14.9	3	14.3 5.4; 23.2	10.0 [10 - 20]	8.8; 15.7	14.1 7.2; 21.1	10.0 [10 - 20]	9.7; 14.6
*Ascaris suum*	27	10.6 6.8; 14.4	6	12.7 4.2; 21.2	3540 [0 – 10,740]	0; 92,416	8.1 2.6; 13.5	953 [0 - 239]	0; 2,969
*Trichuris suis*	22	8.7 5.2; 12.1	4	12.7 4.2; 21.2	25 [0 - 10.7]	0; 83	20.2 0; 4.8	15 [0 - 0.7]	0-79
*Cystoisospora suis*	1	0.4	1	–	–		-	–	
*Strongyloidesransomi*	1	0.4	1	–	–		–	-	

^a^A total of 36.1% of the examined wild boars were not included in the calculation of excretion per age

**Fig. 2:**
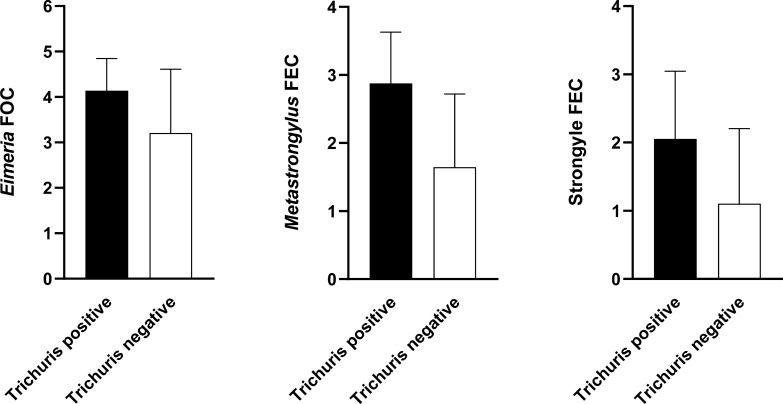
Log-transformed *Eimeria* spp. faecal oocyst counts (FOC) and, *Metastrongylus* sp.- and strongyle faecal egg counts (FEC) according to the *Trichuris suis* infection status (error bars represent the standard deviation)

Wild boars positive for *T. suis* had significantly higher *Metastrongylus* sp. FEC (P < 0.0001), *Eimeria* spp. FOC (P = 0.0024), and strongyle FEC (P < 0.0001) compared to wild boars negative for *T. suis* infection (Fig. 2). *Ascaris suum* FECs were not significant different between *T. suis* positive and negative wild boars. *Metastrongylus* sp. were significantly more prevalent in the young wild boars (P < 0.0001) compared to the adult animals (Table 3. Age related differences in the prevalence were not detected for any of the other parasites.

## Discussion

In this study, we investigated 255 Danish wild boars for bacteria and parasites relevant for the public health and the pig production. This is the first study to document these infections in wild boars in Denmark, which prevents comparison of our results with historic Danish data.

The prevalence of *A. suum* (10.4%) was consistent with the prevalence in wild boars in northern Iran (4.8%) and Estonia (9.0%) [45, 46], while in studies from Turkey and western Iran, *Ascaris* spp. were absent [33, 47], and yet others recorded a higher prevalence (Italy, 88%) [48]. The differences in prevalence may reflect the different age composition of the studied populations. Age-related resistance against *A. suum* is recognized [49]. Different sampling seasons and diverse environmental conditions such as e.g. presence or absence of agricultural fields applied with slurry from domestic pigs may also contribute to the different *A. suum* prevalences reported from various countries. *Ascaris suum* infection is relatively common in domestic pigs [50], but the parasite is mainly important due to the economic losses caused by liver condemnations and the need for anthelmintic treatment. The long-lived *A. suum* eggs is a great challenge for outdoor pig production and even a 2–3 year pasture rotation programme may be insufficient to avoid infection since the eggs can remain infective for up to 7 years [51]. In this study, all samples were collected during the hunting season (October to January) corresponding to late autumn and winter. There may be seasonal variations in the egg/oocyst excretion, as development of most parasite species are temperature-dependent. For example, *A. suum* eggs only embryonate at temperature >14.5 °C, equalling late spring and summer in northern Europe [52]. With a prepatent period of 6–8 weeks [53], a peak in egg excretion might occur during autumn and winter compared to the rest of the year. This also applies for *T. suis* [52] and *Eimeria* spp.. Since these parasite eggs/oocysts can remain viable for several years [51, 54], seasonal differences in egg excretion in this study was not expected. Our results indicate that due to the zoonotic risk of *A. suum* infection, wild boar hunters should take precautions to avoid infection. The main precaution is to avoid contact with faecal material, since *A. suum* is transmitted through oral intake of embryonated eggs, which may stick to the carcasses.

Besides *A. suum*, eight parasite genera were identified in the study, including the zoonotic *T. suis* and *Metastrongylus.* The predominant species were *Eimeria* spp. (92.5%) and *Metastrongylus* sp. (79.5%), which were identified from all sampled enclosures. Wild boars can harbour several *Metastrongylus* species, including *M. asymmetricus, M. confusus*, *M. elongatus*, *M. apri*, *M. pudendotectus* and *M. salmi* [55–57]. Of these, *M. elongatus* and *M. salmi* have been identified in humans as well as wild boars [41, 58]. Metastrongylids were not identified to species level, and it is therefore unknown if zoonotic species were present. Metastrongylids have been absent from Danish domestic pigs for decades [59, 60], while historic data for wild boars is non-existent. Metastrongylids are widespread in European wild boars with prevalences of 28.8–60.0% [34, 48, 61], considerably lower than in our study (79.5%). One important exception though, is a prevalence of >80% reported in wild boars from the Estonian Island Saaremaa [45]. The high prevalences reported in this part of Estonia and in Denmark could result from accumulation of eggs in the soil when wild boars inhabit isolated areas, consequently increasing the infection rate in the intermediate hosts. Nagy et al. [62] showed significantly higher *Metastrongylus* sp. prevalence and infection rate in earthworms in enclosures compared to areas inhabited by free-ranging wild boars. Likewise, feeding sites seemingly constitute a highly infectious reservoir for *Metastrongylus* sp. in wild boars as egg-containing faeces accumulate and increase the infection rate in earthworms significantly compared to other areas [62]. This was supported by a Spanish study, where feeders were built on concrete bases and *Metastrongylus* sp. prevalence in wild boars was independent of feeder density [63].

Hunters reported high piglet fatality rates in two enclosures during preceding years in our study. The high mortality might be associated with *Metastrongylus* sp., and *Eimeria spp.,* considering the high prevalence. In domestic pigs, infections with metastrongylids can be exacerbated by co-existing factors such as secondary bacterial and viral infections [64]. In our study, we observed significantly higher metastrongylid egg counts in wild boars concurrently infected with *T. suis* compared to *T. suis*-negative wild boars. This finding indicates that *T. suis* infection might have the same exacerbating effect on *Metastrongylus* infection in wild boars as bacteria and viruses. Likewise, wild boars positive for *T. suis* infection had significantly higher *Eimeria* spp. FOC and strongyle FEC demonstrating that several parasitic infections in wild boars can be exacerbated by co-infection with *T. suis* or vice versa; wild boars infected with other parasites are more likely to be infected with *T. suis.* However, the study design does not allow us to conclude further.

*Eimeria* spp. infections were also substantially more prevalent (92.5%) in this study than in other studies (7.5–64.2%) [34, 61, 65, 66]. The *Eimeria* spp. oocysts are environmentally resistant and can survive in the environment for a long time, subsequently leading to accumulation of infective oocysts in enclosures. Thus, the high *Eimeria* spp. prevalence in Danish wild boars could (Table 3), as for *Metastrongylus* sp., result from restricting the wild boars in enclosures, and from the close contact between wild boars at the permanent feeding sites.

Although the meat-borne nematode *Trichinella* spp. was absent in the examined wild boars, total absence of *Trichinella* spp. in wild boars in Denmark cannot be proven based on our results, as the prevalence may be very low. A Danish study from 2000, identified three *Trichinella* spp. positive red foxes (0.1%) out of 3133 shoot in 1995–1996 [29]. However, no *Trichinella* spp. infections have been demonstrated in domestic pigs in Denmark since 1930, and for more than 50 years, no autochthonous human *Trichinella* spp. infections have been diagnosed in Denmark. This is in contrast to the neighbouring countries (Germany and Sweden), where *Trichinella* spp. positive wild animals occur [67–69]. The newly built fence along the German–Danish border (finished in 2019) can possibly decrease the risk of *Trichinella* spp. positive wild boars entering Denmark from Germany.

All the examined wild boars tested negative for *B. suis.* However, *Brucella* spp. have been reported in wild boars in other European countries such as Germany, Sweden, Italy, Latvia, and Switzerland [70–72]. In Germany, Al Dahouk et al. [73] found that 22% of wild boar sera were *Brucella* sp*.* seropositive, while Swedish wild boars were sero-negative for *Brucella* sp. in 2013–2015 [74]. The brown hare (*Lepus europaeus*) may become infected with *B. suis* biovar 2 [75] as observed for two Danish brown hares that were diagnosed with *B. suis* biovar 2 in 2002 [76]. Brown hares can thereby act as reservoir for wild boar infection. To our knowledge, brucellosis in Danish brown hares has not been documented since 2002, and it is currently unknown if Danish brown hares constitute a risk for transmission of brucellosis to wild boars.

All wild boars examined in this study tested negative for MRSA. However, MRSA is frequently diagnosed in Danish domestic pigs. In 2018, the screening for MRSA in Denmark showed 20% of the organic pig herds and 89% of the conventional pig farms were positive for MRSA [77]. Outdoor access, lack of antimicrobial usage, and the feed composition was suggested as influencers of low MRSA prevalence in organic pigs [78]. Wild boars are all exclusively housed outdoor and not treated with antimicrobials, but MRSA has previously been diagnosed from wild boars in Portugal [79]. Likewise, all examined wild boars tested negative for *Salmonella* spp.. In Danish pig herds, *Salmonella* spp. cases increased substantially in 2010–2013 following low levels in 2004–2009, primarily in piglets [20]. Routine wildlife disease surveillance in 2003–2018 revealed low prevalence of *Salmonella* spp., with none of the positive animals being wild boars [21]. *Salmonella* spp. infections are, however, reported in wild boars in other European countries such as Sweden [18], Switzerland [80], and Germany [81]. Overall, Danish wild boars constitute an insignificant risk for transmission of MRSA and *Salmonella* spp. to domestic pigs or humans. It is more likely that wild boars may acquire these pathogens from the Danish domestic pigs.

## Conclusions

Of the examined pathogens, only gastrointestinal parasites and lungworms were identified from the Danish wild boars. The most prevalent parasites were *Eimeria* spp. (92.3%) and *Metastrongylus* sp. (79.5%), both of which can be transmitted to domestic pigs. Although human infections are rare, *Metastrongylus* species are considered potentially zoonotic. Relatively few (10.6 and 8.7%, respectively) of the wild boars were infected with the zoonotic parasites *A. suum* and *T. suis*. Therefore, Danish wild boars represent a low risk of transmission to humans, while economically important parasites of domestic pigs were highly prevalent in the wild boars.

## Data Availability

The datasets used and analysed during the current study are available from the corresponding author on reasonable request.
